# Supplementary material for: Reference-based QUantification Of gene Dispensability (QUOD)
Source: Plant Methods. 2021 Feb 9;17:18. doi: 10.1186/s13007-021-00718-5 (PMC7871624; doi:10.1186/s13007-021-00718-5)
Supplement: Supplementary file 2 — Additional file 2. Illustration of the different components of QUOD. [file 13007_2021_718_MOESM2_ESM.pdf]

Input

mapping files (BAM)
(one per investigated genome)

structural annotation of the
reference sequence (GFF)

Calculate read coverage per position (I)

Calculate read coverage per gene (II)

Discard genomes with an average coverage below a given cutoff (default=10) (III)

Construct input matrix (IV)

Determine dispensability score for each gene (V)

$$\text{dispensability score (gene g)} = 1 \Big/ \left[\frac{\sum_{n=1}^{N} \left(\frac{\text{average cov. of gene g in genome n}}{\text{average cov. over all genes in genome n}} \right)}{\text{total number of genomes (N)}} \right]$$

Visualize the score distribution (VI)
