# Supplementary material for: Reference-based QUantification Of gene Dispensability (QUOD)
Source: Plant Methods. 2021 Feb 9;17:18. doi: 10.1186/s13007-021-00718-5 (PMC7871624; doi:10.1186/s13007-021-00718-5)
Supplement: Supplementary file 6 — Additional file 6. Comparison of BUSCO analyses for ‘chlorophyta’, ‘brassicales’ and ‘embryophyta’ as reference. [file 13007_2021_718_MOESM6_ESM.pdf]

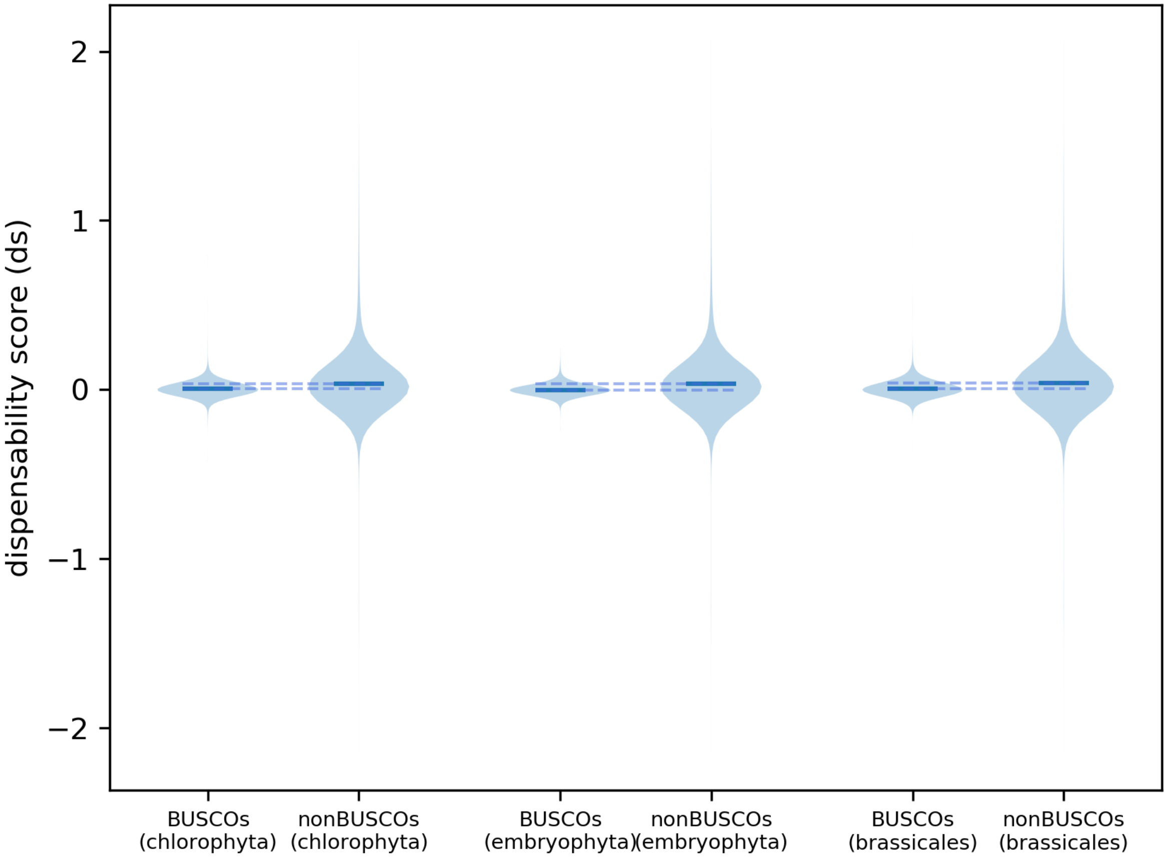

BUSCO statistics

embryophyta	C:98.4%[S:97.6%,D:0.8%],F:0.2%,M:1.4%, n:1614
brassicales	C:98.3%[S:97.0%,D:1.3%],F:0.1%,M:1.6%, n:4596
chlorophyta	C:91.5%[S:56.9%,D:34.6%],F:0.3%,M:8.2%, n:1519

Mann-Whitney U test

chlorophyta	$p \approx$ 4E-61
embryophyta	$p \approx$ 4E-70
brassicales	$p \approx$ 4E-113

Levene's test

chlorophyta	σ^2 (BUSCOs) ≈ 0.021<br>σ^2 (nonBUSCOs) ≈ 3.590<br>$p \approx$ 4E-08
embryophyta	σ^2 (BUSCOs) ≈ 0.005<br>σ^2 (nonBUSCOs) ≈ 3.497<br>$p \approx$ 3E-06
brassicales	σ^2 (BUSCOs) ≈ 0.023<br>σ^2 (nonBUSCOs) ≈ 3.897<br>$p \approx$ 1E-14

Figure S6: Comparison of dispensability scores of BUSCO and non-BUSCO genes using different references (chlorophyta, embryophyta and brassicales). The respective means are represented by the blue lines (dashed lines=extended lines of the respective mean). BUSCO genes show significantly lower scores than non-BUSCO genes for all three reference datasets (Mann-Whitney U test).

Levene's test was used to test for equal variances. The results show that the variances for all reference datasets differs significantly between BUSCO and non-BUSCO genes. Thus, the deviation of the dispensability score from the respective mean is significantly higher for non-BUSCO genes in comparison to BUSCO genes.
