# Supplementary material for: Reference-based QUantification Of gene Dispensability (QUOD)
Source: Plant Methods. 2021 Feb 9;17:18. doi: 10.1186/s13007-021-00718-5 (PMC7871624; doi:10.1186/s13007-021-00718-5)
Supplement: Supplementary file 9 — Additional file 9. Example for lineage specific adaptation. [file 13007_2021_718_MOESM9_ESM.pdf]

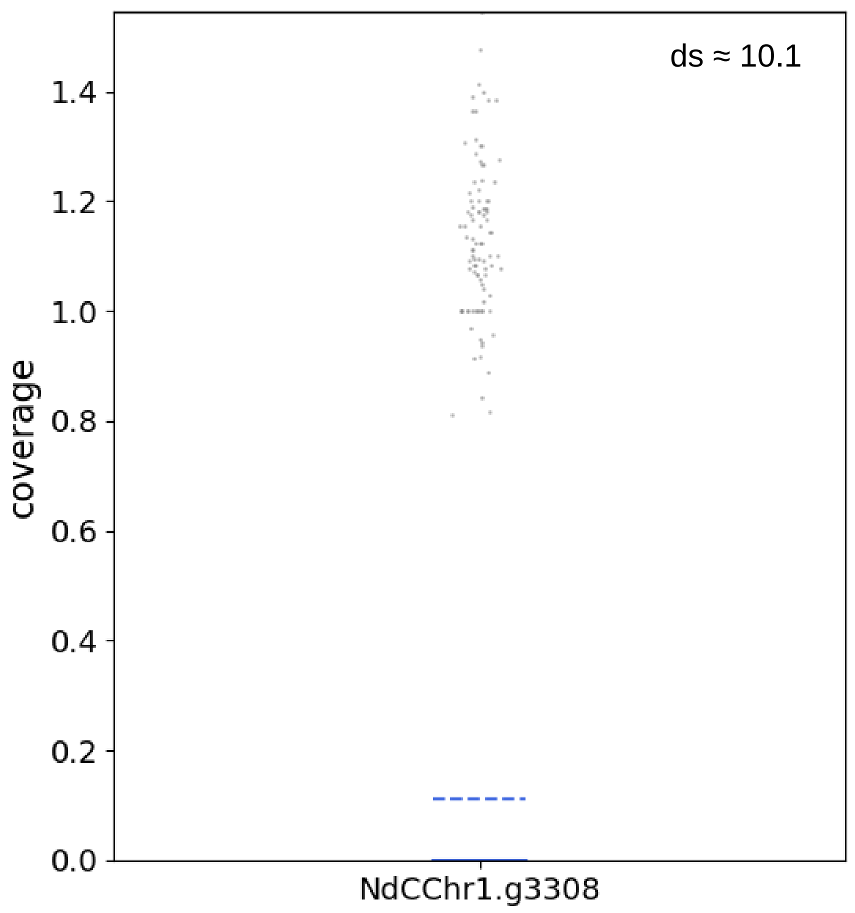

Figure S9: Example for lineage specific adaptation. The gene NdCChr1.g3308 (Nd-1 annotation nomenclature) (ds ≈ 10.1) has zero coverage in 870 accessions (≈ 90%) and is annotated as resistance gene mediating resistance against the bacterial pathogen *Pseudomonas syringae*.
