# Supplementary material for: Reference-based QUantification Of gene Dispensability (QUOD)
Source: Plant Methods. 2021 Feb 9;17:18. doi: 10.1186/s13007-021-00718-5 (PMC7871624; doi:10.1186/s13007-021-00718-5)
Supplement: Supplementary file 11 — Additional file 11. Correlation of the average coverage per gene using three different read mappers: BWA-MEM, bowtie2 and STAR. [file 13007_2021_718_MOESM11_ESM.pdf]

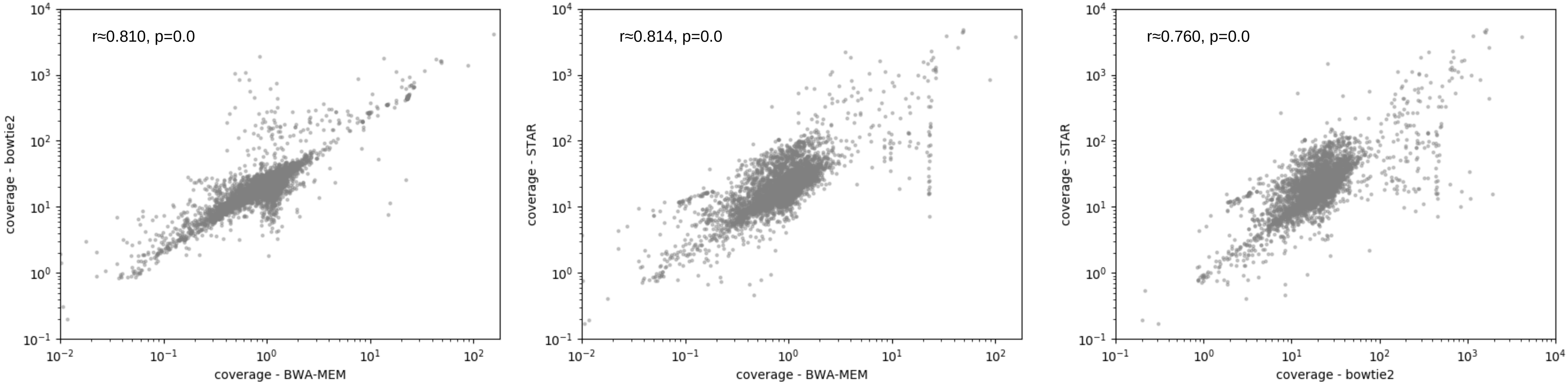

Figure S11: Coverage comparison using different read mappers (BWA-MEM, bowtie2, STAR). Spearman correlation coefficient was used to determine the correlation and the significance of the results. The coverages of the genes using different mappers correlate significantly.
